# Primary hepatic embryonal sarcoma masquerading as metastatic ovarian cancer

**DOI:** 10.1186/1477-7819-7-55

**Published:** 2009-06-23

**Authors:** Peter Kullar, Christopher Stonard, Neville Jamieson, Emmanuel Huguet, Raaj Praseedom, Asif Jah

**Affiliations:** 1 Department of Hepatobiliary and Transplant Surgery, Addenbrooke's Hospital, Cambridge University Hospitals NHS Foundation Trust, Cambridge CB2 2QQ, UK; 2 Department of Histopathology, Chesterfield Royal Hospital NHS Foundation Trust, Chesterfield, Derbyshire DE4 3GJ, UK

## Abstract

**Background:**

Hepatic embryonal sarcoma (HES) is a rare but aggressive primary tumor of the liver occurring most frequently in childhood.

**Case presentation:**

We report a case of a 52 year old woman having previously undergone treatment for ovarian serous papillary carcinoma who subsequently presented with a large solitary mass in the liver. Initially this was presumed to be metastasis from the ovarian primary however, on further examination it was shown to be a primary hepatic embryonal sarcoma.

**Conclusion:**

Primary liver tumors should be considered in differential diagnoses in patients with ovarian cancer who subsequently present with liver tumors. This is particularly important when there is no direct evidence of recurrence of ovarian cancer.

## Background

Hepatic embryonal sarcoma (HES) is rare primary tumor of the liver usually occurring in childhood. It is generally considered an aggressive tumor with a poor prognosis. Approximately 60 cases have been reported in adults. On the contrary, ovarian carcinoma frequently tends to metastasize to the liver [1]. We report a case of a woman who had previously undergone treatment for bilateral ovarian serous papillary carcinoma and subsequently presented with a large solitary mass in the liver which turned out to be a primary hepatic embryonal sarcoma. We also review the current literature, diagnosis and treatment of HES.

## Case presentation

A 52 year old woman presented with pain and a palpable mass in the right upper quadrant of the abdomen 18 months after completing treatment for ovarian carcinoma. She had previously undergone bilateral salpingo-oophorectomy and *en bloc *subtotal colectomy for bilateral high-grade ovarian serous papillary carcinoma infiltrating the sigmoid colon. She also had peritoneal and diaphragmatic seedlings which were debulked concurrently. After the resection, the residual disease was estimated to be less than 0.5 cm^2^. Subsequently, she received 6 cycles of adjuvant chemotherapy with carboplatin and taxol. Her CA125 level which was elevated at 149 U/L prior to resection later returned to 8 U/L (normal range = 0 – 25 U/L). Apart from chemotherapy, there was no past history of exposure to radiotherapy or any carcinogens.

**Figure 1:**
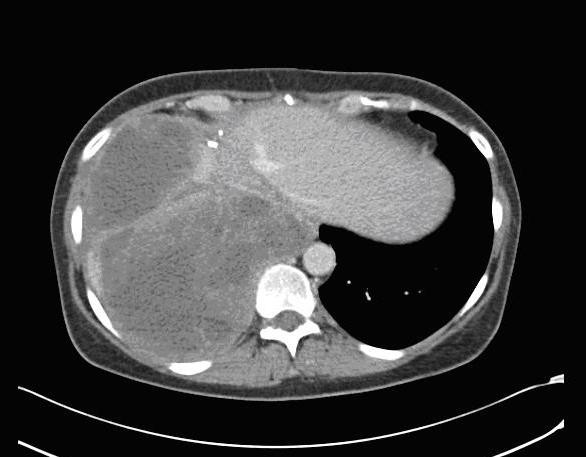
**Computerized Tomography scan of the abdomen, the tumor is shown to be occupying almost the whole the right lobe of the liver**.

During this presentation, the only abnormality on routine hematology and biochemistry was a slightly elevated alkaline phosphatase at 178 U/L (normal range = 35–130 U/L). The CA125 level was within the reference range (7 U/L) and no other tumor markers were assayed. A Computerized Tomography (CT) scan revealed a large heterogeneous mass almost completely replacing the right lobe of the liver (Figure 1). No other abdominal, pelvic or lung lesions were identified. Due to the recent history of ovarian cancer, this lesion was suspected to be a solitary metastatic deposit from the ovarian cancer. The liver lesion was not biopsied in order to avoid any needle tract seeding with malignant cells. In the absence of any extrahepatic disease she proceeded to undergo an extended right hepatectomy. During hepatectomy the tumor was found to be adherent to the diaphragm, a part of which was removed *en bloc*. Post-operatively, she made a good recovery without any complications.

**Figure 2:**
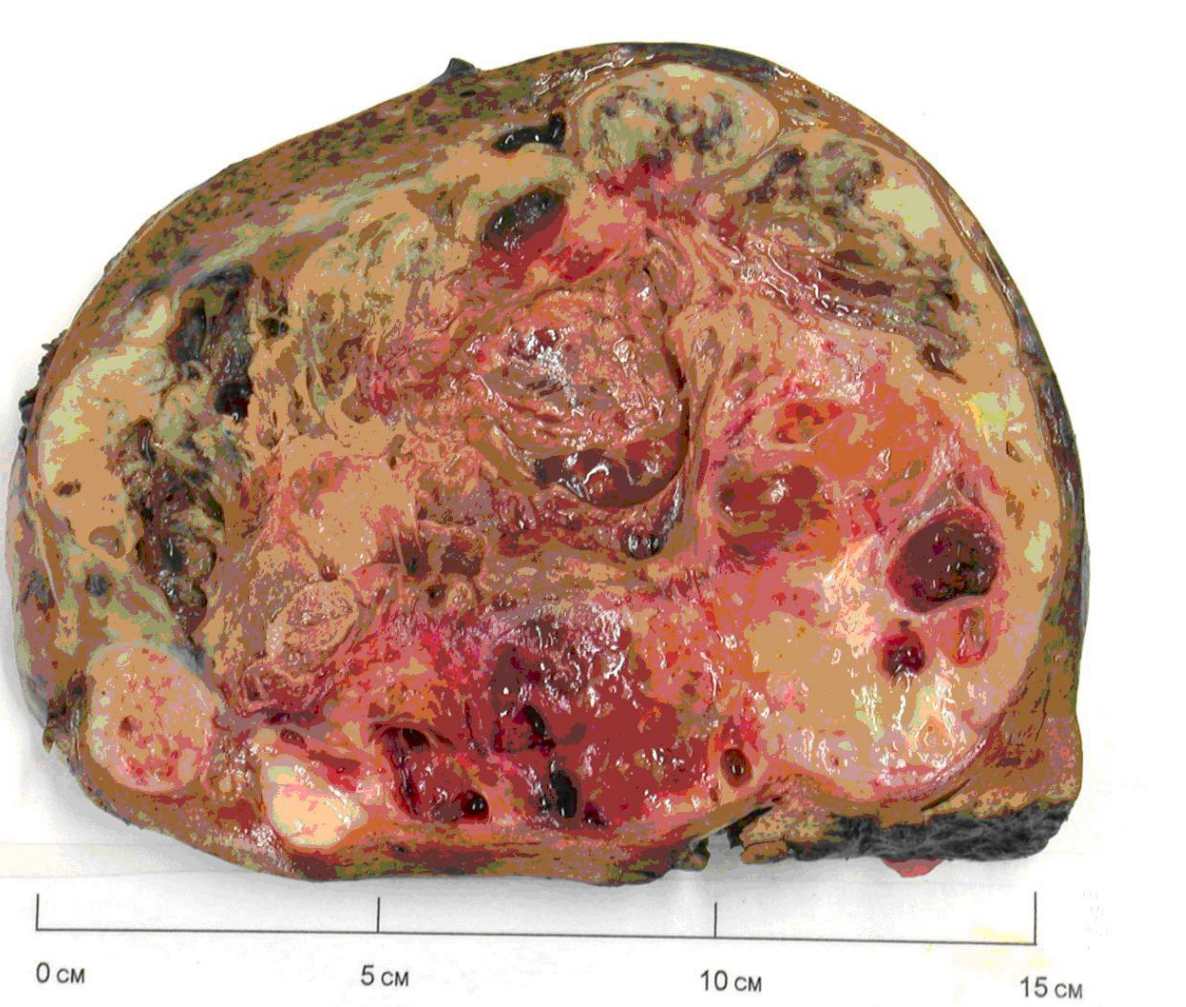
**Cut section of the resected hepatic tumor**.

**Figure 3:**
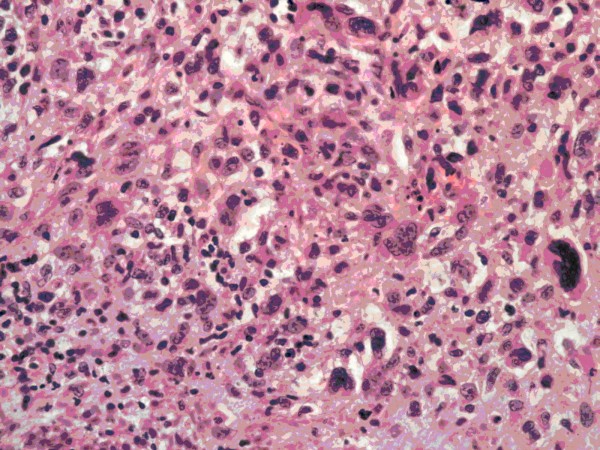
**Representative section of a more cellular area showing plump spindle cells, bizarre giant cell forms and scattered apoptotic bodies**. (Haematoxylin and eosin stain, photographed at ×200 magnification).

Macroscopically, the tumor was a large, solid and cystic mass that included areas of necrosis and cystic degeneration measuring 18 × 12 × 8 cm (Figure 2). Microscopically, it consisted of pleomorphic malignant spindle cells including rhabdoid and bizarre giant cell forms on a chrondromyxoid stromal background (Figure 3) with focal Periodic Acid Schiff-positive cytoplasmic granules. There was no evidence of epithelial differentiation which excluded the possibility of sarcomatoid hepatocellular carcinoma. Immunohistochemical staining was positive for alpha1-antitrypsin but showed no other indicators of differentiation. This contrasted with the serous papillary morphology of the previously resected ovarian tumor that showed no sarcomatoid areas and no rhabdoid differentiation. The liver tumor was therefore judged to be a second primary rather than a metastatic deposit of the resected ovarian carcinoma. The lesion was classified as primary HES on the basis of its morphology and immunohistochemical staining pattern with complete excision noted at the hepatic resection margins.

After remaining well for approximately 6 months she developed progressive shortness of breath and a follow-up CT scan demonstrated a large recurrent tumor contiguous across the right hemithorax and abdomen. A percutaneous biopsy of this mass confirmed the recurrence of the primary hepatic sarcoma. Palliative chemotherapy with ifosfamide and doxorubicin was commenced but had to be stopped after 3 cycles due to severe side effects. She received palliative care for gradual deterioration and died 6 months later.

## Discussion

Ovarian cancer usually presents with widespread intra-peritoneal metastasis [2]. A minority of patients present with aggressive disease manifested by liver, lung or brain metastases [3]. An autopsy study of 428 patients with ovarian cancer reported that over 40% of the patients with ovarian cancer had evidence of liver metastases at the time of death [1]. Amongst the ovarian cancers, the stromal tumors were the most likely histological subtype to metastasize to the liver [1].

Approximately 20% of the patients with epithelial type of ovarian cancer have been reported to have normal levels of CA125 [4,5]. During second presentation of this patient, the liver function tests were slightly deranged but the CA125 level was within the reference range.

Primary liver sarcomas are rare tumors and represent only 0.2% of all primary liver tumors [6]. This group includes a number of different histological types such as angiosarcoma, leiomysarcoma, fibrosarcoma, HES, and malignant fibrous histiocytoma [7]. HES are most commonly reported in a pediatric age group, with the peak incidence between the ages of 6 and 10 years. Only 68 cases have been reported in the adult population [8]. Although the etiology of primary liver sarcomas is unclear, there is an increased risk associated with radiation therapy and high doses of alkylators or anthracyclines [8]. The use of Thorotrast as a radiological contrast medium in the 1950s was associated with a very high incidence of hepatic angiocsarcomas [9]. Our patient had received taxol and carboplatin as adjuvant chemotherapy after resection of ovarian cancer but there is no documented association of these agents with development of primary liver sarcomas.

The presentation of liver sarcomas is usually non-specific with symptoms such as abdominal discomfort, anorexia, fever or weight loss [10]. Hepatomegaly may be present with large tumors and liver function tests may be deranged although frank jaundice is rare [7,10]. The non-specific nature of the presenting symptoms makes clinical diagnosis extremely difficult without imaging or biopsy. Although the lesion can be identified on ultrasonography and CT scan, contrast-enhanced Magnetic Resonance Imaging (MRI) scan is the best imaging modality for characterization of primary liver tumors [11].

There are a number of radiographic features which distinguish HES from mesenchymal and other primary hepatic tumors. The myxoid stroma typical of HES appear as large central areas of hypointense signal on T1-weighted images that have high intensity signal on T2-weighted images. Similarly, in the solid peripheral areas of the mass heterogeneous contrast enhancement is seen on both CT and MRI scans [12].

HES have previously been associated with poor outcome. In 1978 Stocker reported a series of 31 patients with HES with mean survival of less that one year after diagnosis [10]. Although radical surgery remains the mainstay of treatment, recent studies have shown improved survival with radical surgery and use of ifosfamide-based multi-agent chemotherapy. Almogy *et al *reported on a series of 8 primary liver sarcomas including two HES managed with surgical resection and adjuvant chemotherapy [13]. Following a liver resection, one patient with a satellite lesion and a second patient with recurrent HES were treated with chemotherapy which led to tumor shrinkage and enabled a second hepatic resection. There is mounting evidence to suggest that neo-adjuvant chemotherapy would allow down-sizing of HES to be followed by resectional surgery [14,15]. Bisogno *et al *identified 17 children with HES treated with initial conservative surgery and multi-agent chemotherapy followed by second-look surgery for any residual disease [14]. They report 70% (12/17) survival at follow up (ranging from 2.4 to 20 years). Thus, in most cases, the accepted standard treatment would consist of aggressive surgical resection with combination chemotherapy either in neoadjuvant or adjuvant setting.

## Conclusion

In this patient HES masqueraded as a metastatic ovarian carcinoma. This case illustrates that the possibility of a primary liver tumor should be considered in patients with ovarian cancer who subsequently present with liver tumors, particularly when there is no direct evidence of recurrence of ovarian cancer.

## Consent

Written informed consent was obtained from the patient's next of kin for publication of this case report and accompanying images. A copy of the written consent is available for review by the Editor-in-Chief of this journal.

## Competing interests

The authors declare that they have no competing interests.

## Authors' contributions

PK and AJ wrote the manuscript, CS reviewed the histology and contributed to the manuscript, NJ, EH critically reviewed the manuscript and RP performed the hepatectomy. All authors read and approved the manuscript.
